# Age-Associated Tooth Loss and Oral Microbial Dysbiosis in a Mouse Genetic Model of Chronic Nicotine Exposure

**DOI:** 10.3389/fimmu.2020.575200

**Published:** 2020-10-07

**Authors:** Scott W. Rogers, Elizabeth J. Myers, Lorise C. Gahring

**Affiliations:** ^1^Salt Lake City Veterans Affairs Medical Center: Geriatrics, Research, Education and Clinical Center, Salt Lake City, UT, United States; ^2^Department of Neurobiology, University of Utah School of Medicine, Salt Lake City, UT, United States; ^3^Department of Internal Medicine, Division of Geriatrics, University of Utah School of Medicine, Salt Lake City, UT, United States

**Keywords:** nicotinic receptor alpha7, mouse, microbiome, tooth loss, aging

## Abstract

Nicotine acts as a potent modulator of normal cellular responses through the nicotinic acetylcholine receptor subtype alpha7. In a mouse genetic model of alpha7 receptor dysfunction, alpha7^E260A:G^, 85 percent of 18 month-old mice exhibit an age-associated spontaneous loosening or complete loss of 3rd molars that was not present in the control mice. The adjacent soft tissues appeared largely unaffected. Further analysis including micro-CT revealed evidence of bone loss surrounding the 3rd molars with areas of cavitation and/or sponge-like (cancellous) bone remodeling in the mandible. The mandible microbiome was examined using 16S-rRNA sequencing. The results show the alpha7^E260A:G^ oral microbiome included increased landscape complexity indicative of dysbiosis, and a significant increase of some bacteria, particularly *Staphylococcus*. These results suggest that normal alpha7 function plays a relevant role in maintaining normal gene expression and oral microbiome stasis. Consequently, this mouse model suggests there are consequences to ongoing alpha7 receptor dysfunction and oral health, as can occur from chronic exposure to nicotine as expected from electronic nicotine delivery systems (ENDS or “vaping”), that may not be seen until older age.

## Introduction

The response to nicotine is promoted through targeting and interacting with a variety of genetically-related nicotinic acetylcholine receptors (nAChRs) whose function varies dependent upon the individual receptor subunit composition and where they are expressed [e.g., (1–3)]. In the CNS this interaction leads to addiction whereas in peripheral systems a variety of nAChRs of differing pharmacological and functional properties impart highly specific effects on an equally diverse variety of cell types [e.g., (1–3)]. A particularly prominent role in nicotine's effects is imparted through the nAChR subtype alpha7 (α7) whose central role in immune and other peripheral responses is both well-documented and the subject of modulation for therapeutic applications (4–6). It is also expressed by autonomic neurons, keratinocytes, immune cells (particularly macrophages), endothelium, epithelial cells and even highly specialized cells such as ameloblasts (3, 7–10). However, how the nicotine–α7 interaction modulates peripheral cellular responses and especially in the immune system in response to inflammatory processes is not straightforward and mechanistically distinct from CS (10–13). First, the α7 receptor couples to signaling mechanisms in part through its large receptor calcium current that, in addition to the normal sodium/potassium currents, is sufficient to modulate intracellular signaling cascades and transcriptional networks (3, 14). Second, α7 is extraordinarily sensitive to desensitization, a property that closes the receptor ion channel in response to ongoing agonist exposure to agonist, such as nicotine (3, 14). In effect, chronic nicotine exposure can in some cases functionally and phenotypically resemble the effects of a non-functional receptor or those exposed to an antagonist (1). Thus, both activation and desensitization are part of how nicotine will modulate cell-specific responses to exogenous agents, especially in compartments such as the lung or oral cavities where contact with inhaled nicotine is most concentrated. To better understand the importance of these signaling properties to cell response, a mouse was designed in which a point mutation was introduced into the α7 receptor to compromise the calcium ion component of α7 signaling [α7^E260A:G^; see Enioutina et al. (10)] while retaining normal expression. The α7^E260A:G^ mouse has proven particularly applicable in resolving the effects of nicotine-α7 interaction on local cellular responsiveness during a variety of processes ranging from tooth and enamel development to allergen response mechanisms by both macrophages and epithelial cells in the lung (8, 10, 13, 15). It has also proven valuable in distinguishing receptor specific signaling mechanisms from those imparted by cigarette smoke (10, 11).

During the course of these studies it was noted that the 3rd molars in the mandible of older α7^E260A:G^ mice became extremely loose, and they could be easily displaced using light pressure from a metal probe or they had been lost previously. This was rare in the control mice (only one case identified). An assessment of pro-inflammatory gene expression status suggested no major changes between affected mice and control mandibular inflammatory gene expression. Additional screening of the mandibular microbiome using 16S-rRNA sequencing revealed alterations to the α7^E260A:G^ microbiome landscape including altered complexity, bacterial abundance and the disproportionate increase in members, most notably *Staphylococcus*. These results suggest that normal α7 function provides an extended and diverse role in oral health that is relevant to maintenance of local gene expression and microbiome stasis. This also raises a concern as to the consequences that inhaled nicotine, as is currently being experienced by regular users of electronic nicotine delivery systems (ENDS), could have on normal receptor function to impart changes to long-term oral health.

## Methods

### Animal Ethics Statement

Mice for this study were maintained and used in accordance with protocols approved in advance by the Salt Lake City VA Medical Center IACUC (A17/09) and the Institutional Animal Care and Use Committee at the University of Utah (Protocol Number 15-12001). In all cases animals were maintained according to the Guide for the Care and use of Laboratory Animals of the National Institutes of Health.

### The α7 Reporter Mouse Lines

The construction and characterization of the α7^G^ and α7^E260A:G^ mice has been reported in detail (8, 10, 11, 13, 16). Briefly, the α7^G^ harbors a bi-cistronic IRES-tauGFP reporter cassette introduced using the precision of homologous recombination into the 3′ end of *Chrna7* to identify gene transcription using GFP. To modify α7 function, homologous recombination was applied to convert the pore-lining glutamate residue (E260) from the calcium favoring glutamate to an alanine (E260A) in the α7^G^ background (α7^E260A:G^) as described in detail previously. Mice were sex and age-matched and housed at 4 or 5 animals per cage and received standard mouse chow and water ad labitum as described in the prior studies.

### Tooth Preparation

Teeth were defleshed from mandibles of euthanized mice following removal and gentle stripping of soft tissue and placement in commercial grade sodium hypochlorite solution (8). After 24–48 h at room temperature, the skeletal pieces were removed, rinsed in distilled water and air-dried.

### Micro-Computed Tomography (micro-CT) Analysis

Euthanized mice were scanned using a Quantum GX micro-CT Imaging system (Perkin-Elmer). Imaging files were examined using the ImageJ software package (NIH) to generate 2D or 3D reconstructions as before (8).

### RNA Extraction and Quantitative PCR-Array Analysis

The mandibles from 6 α7^G^ or 6 α7^E260A:G^ mice were removed, placed on ice, and thoroughly cleaned of soft tissue under a dissecting microscope. The tissue remaining (proximal incisors, molars and surrounding bone) from each individual was combined, macerated and RNA extracted using the TRIZOL method (Qiagen). Samples were then subjected to analyses using Qiagen RT^2^ Profiler PCR Arrays (PAMM-011E) per instructions.

### Microbiome Analysis

Microbiome analysis followed standard methods (17, 18). Bacterial DNA library preparation and 16S rRNA pyrosequencing of the V3-V4 region was performed (SeqMatic, Freemont CA.), the sequences de-multiplexed and homopolymers or sequences of <300 or >600 bases were removed. The data were analyzed and bacteria classified into Operational Taxonomic Units (**OTU**s) using the default settings of the Quantitative Insights Into Microbial Ecology (QIIME2) pipeline (19). Because the mandible sample counts are subject to variability that often is associated with sporadic detection or very low abundance, only those OTUs exceeding background and present in at least 50% of the mice for at least one α7 genotype were included. Statistical significance of a difference required a *p*-value of <0.05 based upon paired Student's *t*-tests between α7^G^ vs. α7^E260A:G^ averaged results.

## Results

### Mice With Deficient α7 Cell Signaling (α7^E260A:G^) Exhibit Age-Associated Tooth Loss

**Figure 1:**
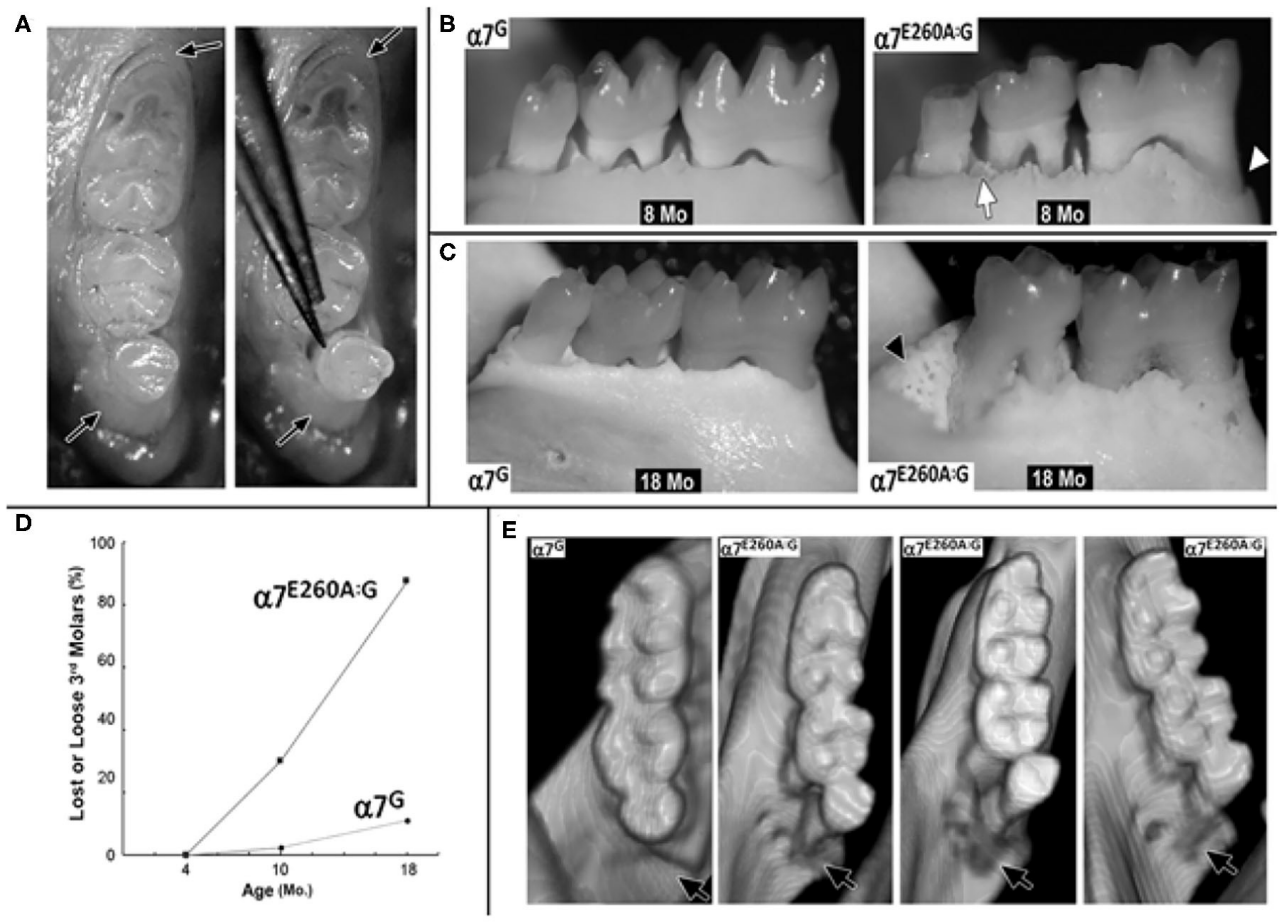
Age-associated increase in 3rd molar looseness and micro-CT images of bone loss in the α7^E260A:G^ mandible. **(A)** Example of 3rd molar looseness before (left panel) and its displacement with light pressure from forceps (right panel). Arrows point to soft tissue surrounding the teeth. **(B)** Defleshed mandibles from 8 month-old (8 Mo) α7^G^ or α7^E260A:G^ mice. Bone loss around the 1st molar root (arrowhead) and porous (cancellous-like) appearance of bone surrounding teeth (arrow). **(C)** Similar images of 18 month-old (18 Mo) mandibles. Note loss of 3rd molar in the α7^E260A:G^ and cancellous appearance of bone replacing the lost molar (arrowhead, 18 mo α7^E260A:G^). **(D)** Quantitation of loose or missing 3rd molars in mandibles of 4 month (*N* = 30 each genotype), 8–10 month (*N* = 40 each genotype) and 18 month old mice (*N* = 18, α7^G^ and *N* = 16, α7^E260A:G^). **(E)** Micro-CT rendered images of the mandibular molars from an α7^G^ mouse (8 mo) or several 8 month old α7^E260A:G^ mice that exhibited a loose 3rd molar. Arrows point to the bone socket and most common site of loss.

As shown in Figure 1A for teeth in the mandible from an 8 month old α7^E260A:G^ mouse the 3rd molars in some of these mice could be easily moved using light pressure as from forceps (Figure 1A). In a few cases, although not common, the 3rd molar was absent. The tissues surrounding the molars were normal and no abnormalities were detected upon gross survey such as loose 1st or 2nd molars nor did this difference extend to maxillary teeth, which were all normal. Thus, the abnormality could be overlooked if not tested for displacement by light pressure. No alterations to the maxillary teeth or superficial palette abnormalities were observed. Examples of the teeth in mice where the loose molar was found, as compared with the control, were examined following being defleshed with outcomes as shown in Figure 1B. Common in the α7^E260A:G^ samples was an apparent reduction in the alveolar bone that in some cases became extensive. The bone surrounding the 2nd and 3rd molars also exhibited substantial porosity suggestive of cancellous-like bone. In these samples, the rostral roots of the molars were often exposed. Given the appearance of these mice, we next examined older mice where these abnormalities were dramatically pronounced and commonly included the complete loss of the 3rd molar. The defleshed samples in Figure 1C show typical appearances of the 18 month-old mice and the marked differences between α7^E260A:G^ mice and the α7^G^ controls. The complete absence of the 3rd molar was common and this was often accompanied by replacement with cancellous-like bone (Figure 1C). This was often accompanied by the exposure of the adjacent 2nd molar root often extending well into the jaw. The bone lining of the mandibular tooth socket were notably uneven and distinguished by extensive porosity. To quantitate this observation, α7 or α7^E260A:G^ mice of both sexes were grouped into three age groups (4–6 months, 8–10 months, and 18–20 months, respectively) and examined for loose or missing 3rd molars. The results in Figures 1B,D show that all teeth in the youngest mice of both genotypes were stable based upon their resistance to displacement from applied pressure. For teeth in the 8–10 month old mice the number of mice exhibiting loose 3rd molars did increase to 6 of 40 in α7^E260A:G^ mice and 1 of 40 in the α7^G^. When this phenotype was examined in the older mice (18 months) the number of loose or missing 3rd molars was increased substantially. In these mice 14 of 16 mandibles (85%) the 3rd molars were loose or absent in the α7^E260A:G^, but only 1 tooth of 18 mandibles examined was loose in the α7^G^ and this required firm and sustained pressure to displace it. There were no differences between male or female mice identified in this phenotype (not shown).

These mice were next examined using Micro-CT and examples of rendered images from 8 month-old teeth are shown in Figure 1E. The appearance of control mice includes the alignment of the teeth and normal bone surrounding the 3^rd^ molar tooth socket. However, in the α7^E260A:G^ mice the 3rd molars were rarely in the same alignment and exhibited an altered register where the 3rd molar tends to be displaced toward the lingual aspect. In addition, there was an apparent loss of bone surrounding and creating the 3rd molar bone socket. This abnormality was not visible if the tooth was present and the mandible not defleshed (as in Figure 1A). In some cases the enlargement of the bone socket was extensive, and the α7^E260A:G^ 3rd molar teeth roots are visible (Figure 1E).

**Figure 2:**
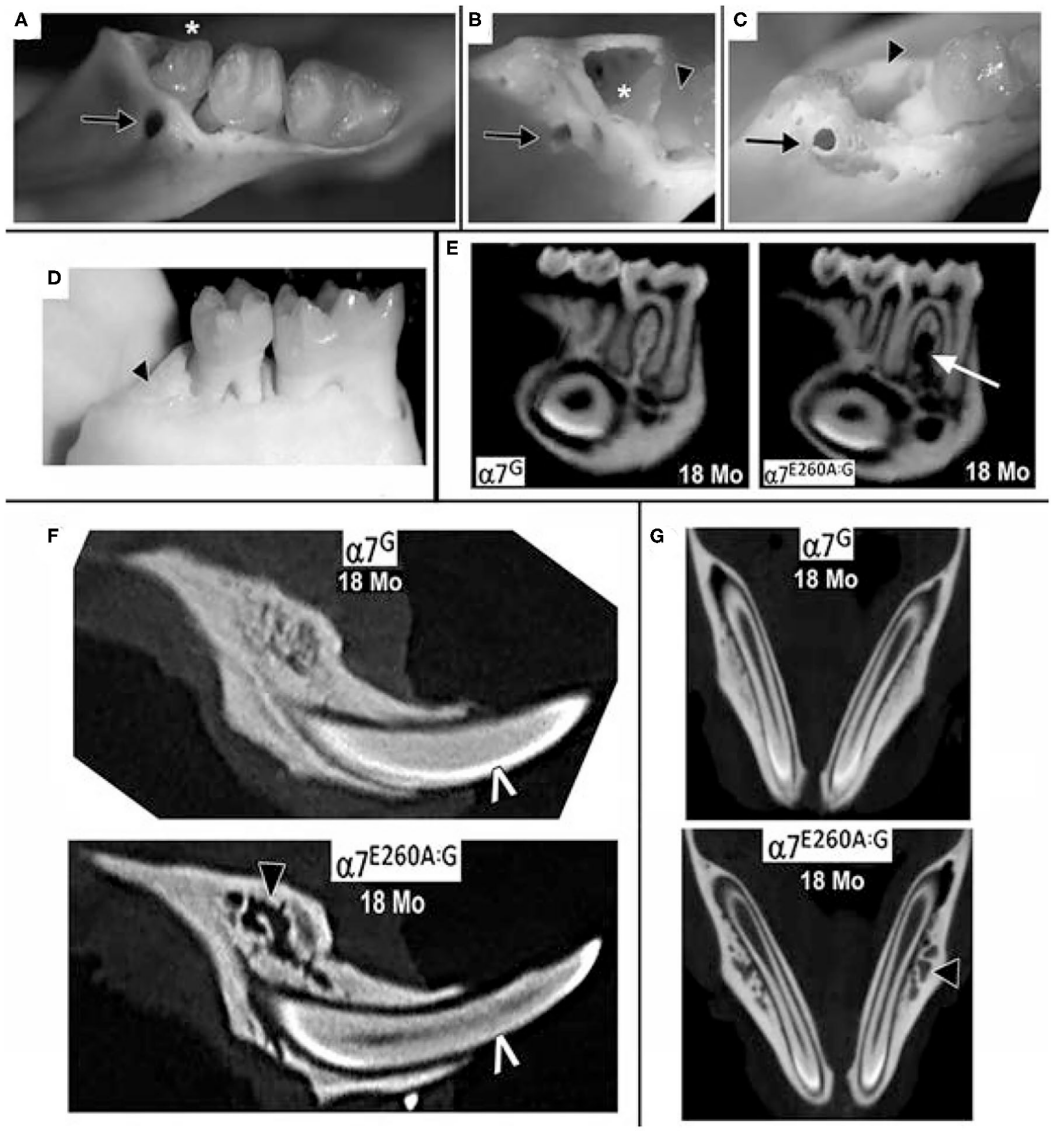
Extensive loss of 3rd molars and local bone loss in the 18 month old α7^E260A:G^ mice mandibles. **(A)** Example of a loose 3rd molar tooth (asterisk) that upon defleshing reveals additional bone abnormities (arrow). **(B)** Another mandible that was missing the 3rd molar prior to defleshing also exhibits bone abnormities (arrow), the socket of the missing 3rd molar (asterisk) and a root of the adjacent 2nd molar (arrowhead). Also, note the porous appearance of the surrounding bone. **(C)** Another example of a bone abnormality (arrow) and the socket of a missing 3rd molar (arrowhead) and exposed root of the 2nd molar. **(D)** An example of a missing 3rd molar where the socket is filled with relatively smooth solid bone-like deposits (arrowhead). **(E)** Sagittal virtual micro-CT view of mandibles from 18 month old mice showing the loss of dense bone material between the roots of the α7^E260A:G^ first molar (arrow). **(F)** A similar sagittal view shows that bone abnormalities extend to other mandible bone regions (black arrow head). The white pointer identifies CT-bright enamel. **(G)** Horizontal micro-CT sections showing the extensive porosity of the bone surrounding the α7^E260A:G^ incisors (black arrow head) when compared to the control.

When the analysis was extended to examine the older α7^E260A:G^ mice, additional irregularities emerged. In some mice, openings in the bone on the lingual aspect that could extend through the bone and into the 3rd molar socket were common (Figure 2A) and these were concurrent with teeth that had fallen out prior to defleshing (Figure 2B). Many of these presented bone with the appearance of being thickened and extended posteriorly along the mandible. In contrast, others lacking the 3rd molar gave the appearance of more fragile bone that was highly cancellous-like porous bone in both mice lacking the 3rd molar (Figure 2B) and in mice where the tooth was present but very loose (Figure 2C). In some mice the cavity created from the loss of the 3rd molar was completely filled (Figure 2D).

Micro-CT images also show that additional changes to the bone in the mandible were observed that differed from controls at 18 months (Figures 2E,F). This included bone loss that was particularly evident between the roots of the first molar and to a lesser extent the second molar (Figure 2E). The bone loss generally extended from the jaw, often near the inferior alveolar nerve passage through the bone and adjacent to the incisor socket, upwards toward the point of contact with the tooth. In some cases the site of inferior alveolar nerve branches at their entry site into the tooth was accompanied by substantial gaps. A closer examination of the 18 month old mandibles and bone structure adjacent to the incisors also showed the alveolar bone could be described as porous in the α7^G^, but in some more advanced cases it was substantially absent in the α7^E260A:G^ mice (Figure 2F). Such differences were also apparent at 8 months, but these deficits tended to be weak and more sporadic. The extensive porosity often extended to the lateral side of the incisor in older α7^E260A:G^ mice and it was not observed in control mice (Figure 2G). Overall this shows there is substantial loss and/or remodeling to the mandible alveolar bone of the older α7^E260A:G^ mice compared to the controls.

### Alterations to the Local α7^E260A:G^ Mandibular Microbiome

The alveolar bone loss observed in the α7^E260A:G^ resembles several other animal models (20–23) and conditions in humans (24, 25), and these are often associated with local increased inflammation and/or infections including the enhanced expression of pro-inflammatory genes. Normal α7 function is associated with anti-inflammatory effects and is most often associated with decreased expression of major inflammatory genes (1–3). Consequently, it is not unexpected that the α7^E260A:G^ mouse exhibits dysregulated responses to inflammatory challenges including allergens and bacterial lipopolysaccharides [LPS; (10, 13)]. To examine this a preliminary measurement of inflammatory gene expression was conducted using RT2-PCR inflammatory gene microarrays [see Enioutina et al. (10), Gahring et al. (26), Gahring et al. (13)]. Comparisons of 3 control mouse mandibles with similar samples from the age-matched α7^E260A:G^ mice failed to identify significant differences in major genes associated with the inflammatory response (e.g., Tnf-α or Il-1β). This suggests that in the older mice the robust modulation of the pro-inflammatory expression usually associated with α7 is either absent in the mandible or not present in the older mice.

**Figure 3:**
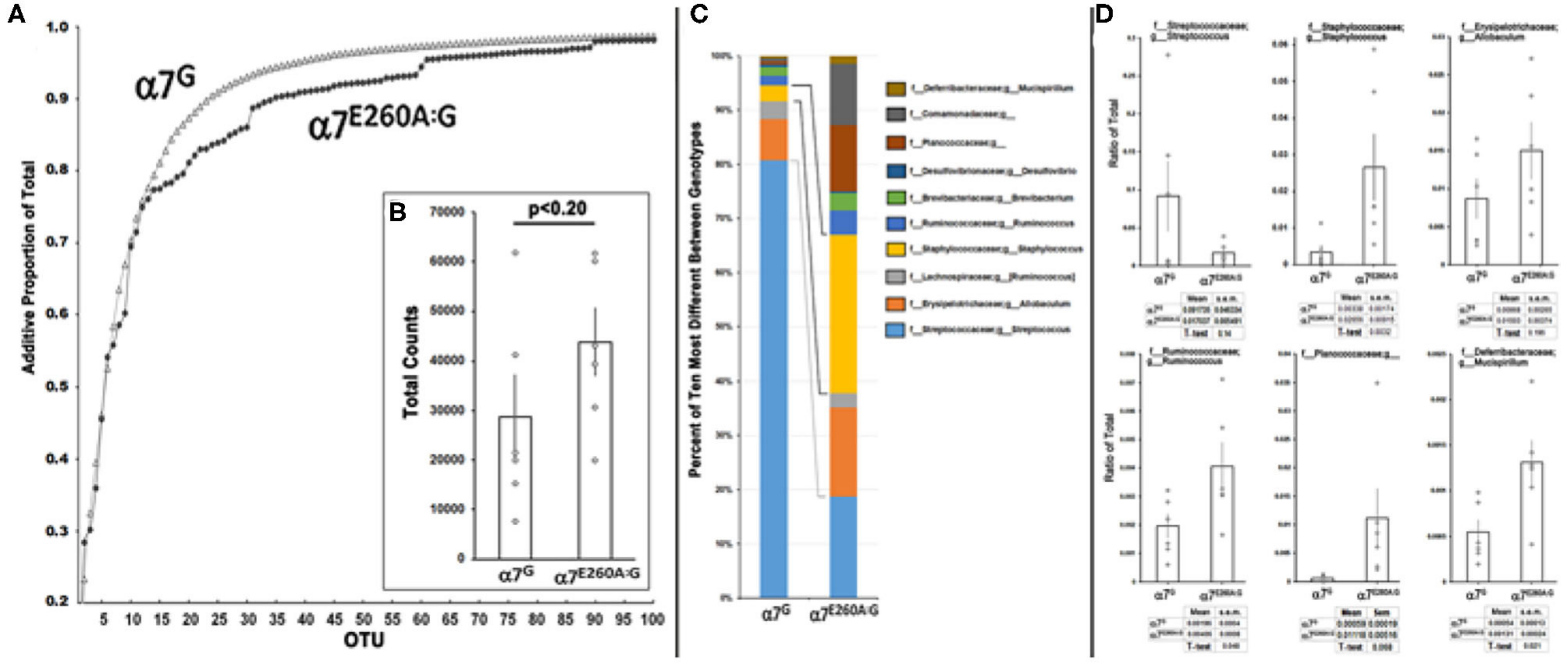
Microbial diversity between α7^G^ and α7^E260A:G^ mandibular samples. Mandibles of six mice from the indicated α7 genotypes were prepared, 16S RNA sequencing conducted and the operational taxonomic units (OTU) assigned to the level of genus using the QIIME2 pipeline (Methods). **(A)** Rarefaction curves show the additive total percentage for the 100 most abundant OTU that were assigned to family or family + genus for α7^G^ (open triangles) and the corresponding value in the α7^E260A:G^ (filled circles). **(B)** Total average bacterial counts for each sample. Error bars are the s.e.m.; p is the result of a standard paired *t*-test. **(C)** Relative percentage for the top 10 most different OTUs between α7 genotypes and assigned to a terminal family or genera as labeled. The lines identify notable differences between the relative proportion of *Streptococcus sp*. (light blue) and *Staphylococcus sp*. (yellow). **(D)** Quantitative comparisons for 6 of the most abundant and different OTU between genotypes as a ratio of their abundance in the total MB landscape. The mean and s.e.m. for each sample is listed and the results of a standard *t*-test that compares the samples.

Therefore, the possibility that these abnormalities could be associated with an altered (dysbiotic) microbiome (MB) landscape was examined (Figure 3 and Methods). For this measurement the mandibles from six each of α7^G^ or α7^E260A:G^ mice were removed aseptically, extensively rinsed with saline, manually defleshed and macerated. The 16S rRNA was then enriched, sequenced and the bacteria were classified into Operational Taxonomic Units (OTU; see Methods). The MB complexity (Figure 3) was then calculated. To do this, the OTU that were present in four or more of the six mice tested and could be assigned to the level of genus in at least one of the α7 genotypes were the focus of the study. This left 153 OTU assignments that accounted for approximately 99 percent of the entire MB landscape for each genotype. The 70 most abundant OTU were then ordered according to the relative abundance in α7^G^ and progressively summed as in Figure 3A and see Table 1. The curves exhibit similarity in abundance among the first 12 most abundant OTUs that account for ~70% of the total MB complexity. These include the abundant S24-7 family members as well as those from Streptococcaceae, Cualobactereacea, Mycoplasmataceae, and Enterobacteriaceae. At this point the α7 genotypes diverge to reveal greater complexity in the α7^E260A:G^ MB landscape. Approximately 95 percent of the MB landscape is achieved in the α7^G^ from the 35 most abundant OTU whereas in the α7^E260A:G^ this required 60 OTU. This shows that in addition to a trend toward greater total abundance, there is also increased MB diversity in the α7^E260A:G^ mandible composed primarily of those OTU of mid-range to lower abundance. In contrast to the gastrointestinal tract where increased biodiversity is viewed as desirable, in the oral cavity robust biodiversity is more associated with susceptibility to pathology (27–29). Total OTU assignments exhibited a non-significant trend that for the α7^G^ were 28,817 counts (8,442 s.e.m.) compared to 43,900 counts (6,894 s.e.m.) for the α7^E260A:G^ (*p* < 0.20; Figure 3B).

**Table 1:** The 70 most abundant OTU from α7^G^ and α7^E260A:G^ mandibles.

**OTU (L6)**	Count^*^		**a7G**		**a7E260A:G**		***t*-test**
	**a7G**	**a7E260A:G**	**Mean**	**s.e.m**.	**Mean**	**s.e.m**.	
f__S24-7;g__	6	6	2.33E-01	4.47E-02	2.85E-01	4.12E-02	0.42
f__Streptococcaceae;g__Streptococcus	6	6	9.17E-02	4.63E-02	1.70E-02	5.49E-03	0.14
o__Clostridiales;f__;g__	6	6	7.03E-02	1.88E-02	5.80E-02	1.15E-02	0.59
f__Caulobacteraceae;g__	6	6	6.64E-02	3.08E-02	9.60E-02	2.37E-02	0.46
f__Mycoplasmataceae;g__	6	6	6.42E-02	2.58E-02	8.56E-02	3.42E-02	0.63
f__Enterobacteriaceae;g__	6	6	5.72E-02	3.94E-02	1.71E-02	3.80E-03	0.33
f__Enterococcaceae;g__Enterococcus	6	6	5.19E-02	2.45E-02	2.76E-02	8.56E-03	0.37
f__Carnobacteriaceae;g__Carnobacterium	6	5	3.45E-02	2.39E-02	1.63E-02	4.37E-03	0.47
f__Lactobacillaceae;g__Lactobacillus	6	6	3.31E-02	2.18E-02	9.23E-02	7.62E-02	0.47
f__Rikenellaceae;g__	6	6	3.14E-02	1.36E-02	2.01E-02	2.79E-03	0.44
f__Helicobacteraceae;g__	6	6	2.45E-02	8.02E-03	3.47E-02	1.11E-02	0.47
f__Pasteurellaceae;g__Actinobacillus	6	6	1.79E-02	1.08E-02	1.14E-02	8.80E-03	0.65
f__Bacteroidaceae;g__Bacteroides	6	6	1.76E-02	5.86E-03	1.27E-02	2.36E-03	0.46
f__[Tissierellaceae];g__Finegoldia	6	5	1.71E-02	1.65E-02	1.23E-03	8.95E-04	0.36
f__Pasteurellaceae;g__Aggregatibacter	6	6	1.67E-02	9.84E-03	6.95E-03	5.84E-03	0.41
f__[Weeksellaceae];g__	6	6	1.62E-02	1.29E-02	1.80E-03	9.19E-04	0.29
f__Lachnospiraceae;g__	6	6	1.10E-02	4.71E-03	8.11E-03	1.38E-03	0.57
f__[Odoribacteraceae];g__Odoribacter	6	6	9.49E-03	5.76E-03	4.52E-03	1.13E-03	0.42
f__Erysipelotrichaceae;g__Allobaculum	6	6	8.69E-03	2.65E-03	1.50E-02	3.74E-03	0.20
f__Prevotellaceae;g__Prevotella	6	6	8.03E-03	2.60E-03	1.03E-02	1.84E-03	0.50
f__[Paraprevotellaceae];g__[Prevotella]	6	6	7.90E-03	2.20E-03	8.45E-03	2.22E-03	0.87
f__[Tissierellaceae];g__Anaerococcus	3	5	7.87E-03	7.52E-03	5.05E-04	2.45E-04	0.35
f__Ruminococcaceae;g__Oscillospira	6	6	6.91E-03	1.26E-03	5.58E-03	1.11E-03	0.45
f__Anaeroplasmataceae;g__Anaeroplasma	6	6	5.37E-03	3.44E-03	2.71E-03	8.63E-04	0.47
o__Bacteroidales;f__;g__	6	6	4.82E-03	1.77E-03	3.30E-03	6.54E-04	0.44
f__Bradyrhizobiaceae;g__	6	6	4.39E-03	2.13E-03	7.01E-03	1.94E-03	0.38
f__Ruminococcaceae;g__	6	6	4.16E-03	7.50E-04	4.03E-03	6.66E-04	0.90
f__Methylobacteriaceae;g__Methylobacterium	6	6	3.85E-03	1.76E-03	5.31E-03	1.39E-03	0.53
f__Lachnospiraceae;g__[Ruminococcus]	6	6	3.74E-03	1.06E-03	2.36E-03	5.86E-04	0.28
f__Staphylococcaceae;g__Staphylococcus	6	6	3.38E-03	1.74E-03	2.66E-02	9.15E-03	0.03
f__F16;g__	6	6	3.34E-03	9.25E-04	3.10E-03	7.71E-04	0.85
f__Corynebacteriaceae;g__Corynebacterium	6	6	2.77E-03	9.79E-04	4.66E-03	1.81E-03	0.38
f__Desulfovibrionaceae;g__	6	6	2.11E-03	4.56E-04	2.84E-03	8.38E-04	0.46
f__Ruminococcaceae;g__Ruminococcus	6	6	1.96E-03	4.30E-04	4.07E-03	8.34E-04	0.05
f__Rikenellaceae;g__AF12	5	5	1.93E-03	6.36E-04	1.40E-03	3.41E-04	0.48
f__Alcaligenaceae;g__Sutterella	6	6	1.86E-03	8.01E-04	1.61E-03	5.11E-04	0.79
f__Rikenellaceae;g__Rikenella	5	3	1.82E-03	1.20E-03	4.59E-04	3.18E-04	0.30
f__Brevibacteriaceae;g__Brevibacterium	6	6	1.80E-03	5.03E-04	2.93E-03	7.14E-04	0.22
f__Moraxellaceae;g__Acinetobacter	6	6	1.80E-03	1.48E-03	1.21E-03	7.06E-04	0.73
f__Caulobacteraceae;Other	5	6	1.75E-03	1.16E-03	1.26E-03	2.98E-04	0.70
f__Enterococcaceae;Other	6	6	1.60E-03	6.84E-04	9.62E-04	3.55E-04	0.43
f__Verrucomicrobiaceae;g__Akkermansia	6	6	1.56E-03	6.35E-04	1.20E-03	4.92E-04	0.67
f__Porphyromonadaceae;g__Porphyromonas	6	6	1.55E-03	5.46E-04	1.56E-03	9.96E-04	1.00
f__Helicobacteraceae;Other	5	6	1.42E-03	4.94E-04	3.22E-03	1.42E-03	0.26
o__YS2;f__;g__	5	6	1.21E-03	4.80E-04	1.71E-03	3.52E-04	0.42
f__Ruminococcaceae;Other	6	6	1.08E-03	1.15E-04	1.08E-03	2.41E-04	1.00
f__Turicibacteraceae;g__Turicibacter	5	3	9.63E-04	3.43E-04	5.34E-04	2.84E-04	0.36
f__Micrococcaceae;g__Micrococcus	5	6	8.78E-04	5.93E-04	1.18E-03	4.88E-04	0.70
f__Enterobacteriaceae;g__Enterobacter	5	5	8.62E-04	6.91E-04	1.26E-04	4.82E-05	0.31
f__Lachnospiraceae;g__Coprococcus	6	6	8.52E-04	1.41E-04	7.44E-04	1.70E-04	0.63
f__Chitinophagaceae;g__Sediminibacterium	5	6	7.97E-04	4.37E-04	1.30E-03	3.03E-04	0.36
f__Leuconostocaceae;g__Leuconostoc	4	6	7.43E-04	4.45E-04	7.28E-04	2.21E-04	0.98
f__Listeriaceae;g__Brochothrix	5	6	7.22E-04	3.16E-04	3.68E-03	1.77E-03	0.13
f__Lachnospiraceae;Other	6	6	6.94E-04	2.32E-04	4.21E-04	1.24E-04	0.32
f__Dehalobacteriaceae;g__Dehalobacterium	5	6	6.69E-04	2.29E-04	1.18E-03	3.29E-04	0.23
o__Sphingobacteriales;f__;g__	6	6	6.59E-04	2.74E-04	9.52E-04	5.16E-04	0.63
f__Desulfovibrionaceae;g__Desulfovibrio	6	5	6.33E-04	1.86E-04	2.95E-04	8.98E-05	0.13
f__Propionibacteriaceae;g__Propionibacterium	5	6	5.98E-04	2.77E-04	1.21E-03	2.61E-04	0.14
f__Planococcaceae;g__	6	6	5.96E-04	1.98E-04	1.12E-02	5.17E-03	0.07
f__Comamonadaceae;g__	6	6	5.91E-04	2.27E-04	1.03E-02	6.36E-03	0.16
f__Sinobacteraceae;g__	6	5	5.83E-04	4.66E-04	8.43E-04	3.46E-04	0.66
f__Lachnospiraceae;g__Dorea	5	6	5.77E-04	2.46E-04	5.08E-04	2.40E-04	0.85
f__Deferribacteraceae;g__Mucispirillum	6	6	5.46E-04	1.36E-04	1.31E-03	2.45E-04	0.02
f__Xanthomonadaceae;g__	5	6	5.46E-04	4.37E-04	3.16E-04	1.26E-04	0.62
f__Bacillaceae;g__Bacillus	6	4	5.35E-04	1.62E-04	5.69E-04	3.08E-04	0.92
Unassigned	6	6	5.32E-04	9.24E-05	7.75E-04	1.51E-04	0.20
o__Clostridiales;Other;Other	6	6	5.18E-04	1.33E-04	2.76E-04	7.73E-05	0.15
f__Helicobacteraceae;g__Helicobacter	6	6	4.83E-04	1.72E-04	6.69E-04	2.56E-04	0.56
f__Porphyromonadaceae;g__Parabacteroides	5	5	4.82E-04	2.00E-04	3.57E-04	1.39E-04	0.62
f__Bifidobacteriaceae;g__Bifidobacterium	6	6	4.69E-04	1.19E-04	5.63E-04	2.60E-04	0.75

* *Count = The number of animals from 6 total of each genotype, respectively, in which the OTU was measured*.

A closer examination of the 10 OTU that were most different between α7 genotypes in terms of percentage representation in the total MB landscape is shown in Figure 3C. As is consistent with the complexity analysis results, they represent an overall less abundant class of OTU as they compose <10 percent of the total MB landscape in either the α7^G^ or α7^E260A:G^. Of these, three were statistically different (increased) in the α7^E260A:G^ (*p* < 0.05); *Staphylococcus, Ruminococcus*, and *Mucispirillum* (Figure 3D). The other OTU included some that exhibited very strong trends in content, but also considerable variability among mice, even those of the same cage. This included the most abundant of the OTU in this group, *Streptococcus, sp*., which was strongly decreased in its percentage of abundance in the α7^E260A:G^ relative to the controls, but this did not achieve statistical significance (*p* < 0.14; *N* = 6 each genotype). The α7^G^ distribution of *Streptococcus* resembled bi-modal distribution with three mice exhibiting relatively high abundance and three that were much lower. This distribution appears independent of mice from the same cage, sample group or detected oral pathology (not shown). Ruminococcus and Mucispirillum can be considered as opportunistic pathogens. Some Mucispirillum species of have been associated with intestinal inflammation that favors the production of intestinal IgA (30). Assessment of these possibilities will require additional study to better resolve taxonomic identity. Finally, the family Planococcaceae was particularly favored for expression in the α7^E260A:G^. The reasons for this are unclear although it has been linked as a marker of several disorders including those associated with obesity (31).

In summary, the results show that α7 dysfunctional signaling is accompanied with 3rd molar tooth loss and mandibular bone damage. This is accompanied with increased bacterial landscape complexity and a notable rise in *Staphylococcus* abundance. The results strongly suggest that α7 receptor dysfunction and reduced signaling can lead to age-associated changes of varied severity, which may be manifested differently among older individuals.

## Discussion

Nicotine, when delivered through the more traditional route of cigarette smoking, interacts with nicotine acetylcholine receptors that both leads to addiction, but also changes many physiological processes in peripheral systems. Among these is the prominent and well-documented impact by nicotine on a variety of fundamental processes that includes an impact on modulating inflammatory and immune responsiveness. While many nAChRs are expressed in peripheral systems (1–3, 8, 10, 32, 33), the α7 receptor is prominent for both its substantial impact on the function of those cells, but also its value as a target for therapeutic modulation of inflammation (4–6). One approach to defining the role of α7 in these processes has been through the application of genetic manipulation of individual nAChR subunits to impact receptor function in specific ways. In this case the α7^E260A:G^ mouse has been implemented to specifically define how signaling through the α7 ion channel couples to cell signaling events with consequence on a broad range of immune responses to allergens and bacterial agents (10–13, 26) and developmental process including tooth and enamel development (8). This mouse has the added advantage over genetic ablation due the minimally invasive nature of the missense mutation to the gene and genomic structure and complications that arise due to abnormal receptor expression or even compensation. This approach has aided in establishing the distinct role played by the α7 receptor and how the nicotine-α7 interaction differs from CS [e.g., (12, 26)] or is specific to the phenotype being examined.

Nicotine, in addition to being a receptor agonist, if present at greater concentrations or for longer exposure durations may actually move α7 into a more desensitized or even inactivated state (3, 14, 34). Thus in heavy ENDS users the exposure by cells exposed to the nicotine-vapor will likely compromise α7 signaling in a manner that resembles α7^E260A:G^ dysfunction. Thus diminished signaling of the α7^E260A:G^ in peripheral tissues can model how prolonged ENDS nicotine-vapor exposure as in heavy ENDS users will impact those cells that are reliant upon this mechanisms of signaling. In a recent study (26) we demonstrated that exposure to α7 receptor-specific positive allosteric modulators that relieve the desensitized state are sufficient to restore normal lung alveolar macrophage signaling to an allergen in lungs exposed to aerosolized nicotine or in response to specific pro-inflammatory cytokine treatments *in vitro*. While the loss of the third molar is likely to reflect its more vulnerable size and location the jaw, it will be of interest to see if other teeth are lost with more advanced age. While the α7^E260A:G^ mutation is sufficient to produce this age-associated phenotype, it is unclear if the bone abnormalities are due to an influence by α7 on maintaining normal bone stasis or due to promoting conditions favorable to certain bacteria that in turn modify the local bone structure. In preliminary three point bone fracture studies we observed that femurs of the α7^E260A:G^ are significantly weaker than age-matched controls (not shown). However, it is unclear if this is related to age, or if changes reflect differences in mineral content or local organ cellular functions. The alveolar bone loss also resembles several other animal models (20–23) and conditions in humans (24, 25) that are often associated with local increased inflammation and/or infections including the enhanced expression of pro-inflammatory genes. However, the robust modulation of prominent inflammation cytokines such as Tnf-α and Il-1β by α7, while common in acute challenges to inflammogens (3, 35), appear to be similar to the controls. This could reflect the late stage of measurement when the modulatory role of α7 is absent or overwhelmed by other more robust processes. This can be better examined in mice where the initiation of the process and how exposure to aerosolized nicotine impacts progression can be more precisely controlled and subject to evaluation through application of more comprehensive methods of measurement including RNA-Seq.

In this study, given the apparent absence of a strong inflammatory response, it is unclear as to whether the bone abnormalities are due to an influence by α7 on maintaining normal bone stasis or due to the effects of bacteria on local bone structure. What seems likely is the possibility that physical barriers are compromised to permit access to the bone by opportunistic bacteria that may otherwise be harmless, such as *Staphylococcus*. Such infections in humans are commonly associated with osteomyelitis and even oral infections associated with root canals and the jaw (36–40). An important future study is to confirm this result and focus on defining the mechanism(s) of how α7 function influences the changes leading to this disorder and to local bone loss (e.g., osteomyelitis, bone necrosis, or other). What is clear is that disruption of normal α7 signaling, as is likely in oral tissues subjected to direct nicotine exposure delivered in ENDS, can lead to pleiotropic effects of which some are likely to promote susceptibilities to potential pathological processes of varied severity that may not be manifested until older age.

## Data Availability Statement

The datasets presented in this study can be found in online repositories. The names of the repository/repositories and accession number(s) can be found in the article/supplementary material.

## Ethics Statement

The animal study was reviewed and approved by Salt Lake City VA Medical Center IACUC (A17/09) and the Institutional Animal Care and Use Committee at the University of Utah (Protocol Number 15-12001).

## Author Contributions

SR collected data, wrote the main manuscript text, and prepared figures. All authors contributed to experimental design, data analysis, and reviewed the manuscript.

## Conflict of Interest

The authors declare that the research was conducted in the absence of any commercial or financial relationships that could be construed as a potential conflict of interest.
